# Micronutrient and Nutritional Status of HIV-Exposed and HIV-Unexposed Malawian Infants in the First Year of Life: Assessment of Ferritin, Vitamin A, and D Status and Its Association with Growth

**DOI:** 10.3390/nu15143282

**Published:** 2023-07-24

**Authors:** Marco Floridia, Clementina Maria Galluzzo, Stefano Orlando, Richard Luhanga, Robert Mphwere, Thom Kavalo, Mauro Andreotti, Roberta Amici, Fausto Ciccacci, Maria Cristina Marazzi, Marina Giuliano

**Affiliations:** 1 National Center for Global Health, Istituto Superiore di Sanità, 00161 Rome, Italy; clementina.galluzzo@iss.it (C.M.G.); mauro.andreotti@iss.it (M.A.); roberta.amici@iss.it (R.A.); marina.giuliano@iss.it (M.G.); 2 Department of Biomedicine and Prevention, University of Rome Tor Vergata, 00133 Rome, Italy; stefano.orlando@uniroma2.it; 3 DREAM Program, Community of S. Egidio, Blantyre P.O. Box 30355, Malawi; richardluhanga@gmail.com (R.L.); robertmphwere@gmail.com (R.M.); thomkavalo123@gmail.com (T.K.); 4 UniCamillus, Department of Medicine, Saint Camillus International University of Health Sciences, 00131 Rome, Italy; fausto.ciccacci@unicamillus.org; 5 DREAM Program, Community of S. Egidio, 00153 Rome, Italy; cristina.marazzi@dreamsantegidio.net

**Keywords:** HIV, infant growth, Africa, malnutrition, ferritin, vitamin A, RBP, vitamin D, HEU, HUU

## Abstract

Breastfed Malawian infants from Human Immunodeficiency Virus (HIV)-uninfected and HIV-infected women who received antiretroviral therapy were followed until 12 months of age, allowing us to evaluate plasma levels of ferritin, vitamin A (as retinol-binding protein, RBP), and vitamin D (25(OH)D) at six months, as well as nutritional status and growth between six and 12 months. Ferritin and RBP levels were adjusted for inflammation. The study included 88 infants, 63 of whom were part of a recent cohort (2019–2021) that included 49 HIV-exposed but uninfected (HEU) and 14 HIV-unexposed and uninfected (HUU) infants, as well as 25 infants (all HEU) from an earlier cohort (2008–2011). No differences were observed between HEU and HUU infants regarding micronutrient levels, anthropometric indexes, growth, and rates of stunting, being underweight, or wasting. HEU infants from the earlier cohort, when compared to more recent HEU infants, had significantly worse anthropometric measures at six months and inferior growth between six and twelve months. Overall, ferritin deficiency involved 68.6% of infants, while vitamin A and vitamin D deficiency involved 8% and 1.2% of infants, respectively. Micronutrient deficiencies were not associated with HIV exposure, cohort, stunting, being underweight, or wasting. At six months, stunting, being underweight, and wasting involved 25.0%, 2.7% and 2.8% of infants, respectively, with no differences related to HIV exposure. Ferritin deficiency at six months was associated with inferior subsequent growth. In this small observational study conducted in Malawian infants, no major nutritional gap was observed between HIV-exposed and HIV-unexposed infants, though the study highlighted specific nutritional deficiencies that deserve attention. High rates of stunting and ferritin deficiency were observed in the first year of life in Malawian infants, irrespective of maternal HIV status; a significant association between ferritin deficiency and worse subsequent growth was found. Vitamin A and vitamin D deficiencies were much less frequent. Based on the data observed, nutritional interventions should give priority to the correction of ferritin deficiency and chronic undernutrition.

## 1. Introduction

Nutrient intake, infections, and maternal health have a key role in determining infant health. In low-income countries, sociodemographic factors, malnutrition, and infections, particularly HIV, are strictly interconnected, with each of these three conditions increasing the likelihood and severity of the others, ultimately leading to impaired growth, substantial morbidity, and increased risk of death among infants. Chronic suboptimal nutrition, which is represented by inadequate caloric and/or nutrient intake in utero or post-natal life, usually translates into stunting (low height for age). This condition is associated with several adverse outcomes, which include impaired cognitive development [1], higher risk of overall morbidity [2], and increased occurrence of infections [3], with consequences that are often carried forward in terms of reduced stature and lean body mass in adulthood [4].

Wasting (low weight for length) represents a potentially severe condition caused by infections, malnutrition, or both factors [5], which may require urgent medical and nutritional interventions to revert an acutely increased risk of death [6,7].

Globally, 149.2 million children aged under 5 years old suffered from stunting in 2020, while 45.4 million children suffered from wasting, with the global prevalence of these conditions being 22.0% and 6.7%, respectively [6]. In most African countries, rates of stunting are even higher, commonly exceeding 30% [6], and the concomitant high prevalence of HIV infection may further worsen the clinical effects of the interconnection between infections and malnutrition.

Programs created for the prevention of mother-to-child transmission of HIV have dramatically reduced the number of HIV-infected infants. There is, however, some evidence that maternal HIV infection, irrespective of HIV transmission, may represent an independent risk factor for infant morbidity. HIV-exposed and uninfected infants, compared to HIV-unexposed and uninfected infants, have been reported to show higher rates of infections, such as pneumonia, tuberculosis, diarrhea, sepsis, and fungal infections [8,9,10,11]. Such infections might be favoured not only based on immunological abnormalities linked to maternal HIV or worse maternal conditions, but also based on micronutrient deficiencies and malnutrition. Exploring, in detail, the possible differences between the populations of HIV-exposed and HIV-unexposed infants might, therefore, help us to define specific nutritional interventions for HIV-exposed and uninfected infants, ultimately improving their health.

Low micronutrient levels may predispose infants to different adverse health outcomes. Insufficient or deficient vitamin D levels have been associated with growth impairment and increased susceptibility to infections, diarrhea, tuberculosis, CMV, and malaria [12,13,14,15,16]. Similarly, vitamin A deficiency has been associated with early stunting [17], infections [18], and anemia, with vitamin A supplementations associated with a clinically meaningful reduction in morbidity and mortality in children [19]. The impact of anemia and iron deficiency on child mortality is also known, with an estimated reduction of 24% in risk of death for each 1-g per decilitre increase in haemoglobin [20].

Despite these premises, data regarding the prevalence of micronutrient deficits in African infants are limited and often inconsistent, possibly because of regional differences or variable definitions used. Few studies have also concomitantly evaluated levels of multiple micronutrients, nutritional status, and growth in infants with different HIV exposure status.

In order to provide additional information on this subject, we investigated levels of ferritin, retinol-binding protein (RBP), and vitamin D, together with two acute-phase proteins (APP) that act as inflammation markers (C-reactive protein, CRP; alpha1-acid glycoprotein, AGP) in two groups of breastfed Malawian infants with different HIV exposure status (HIV-exposed uninfected, HEU born to HIV-positive mothers; HIV-unexposed, uninfected, HUU, born to HIV-negative mothers). We also assessed the infants’ nutritional status (stunting, wasting, and being underweight) and the potential impacts of micronutrient deficiency on infant growth in the first year of life.

## 2. Methods

### 2.1. Study Population

This analysis included infants from two cohort studies. Both cohorts were followed within the structure of the Malawian DREAM (Disease Relief through Excellent and Advanced Means) Program of the Community of S. Egidio, which is an Italian faith-based non-governmental organization that offers health services for HIV, TB, and non-communicable diseases in several countries [21,22,23].

The more recent cohort (GF cohort) was followed in a study, which was conducted in 2019–2021, that assessed maternal retention in programs for the prevention of vertical HIV transmission and compared several health parameters in HEU and HUU infants during the first year of life. This cohort enrolled 163 HEU and 72 HUU infants. Maternal inclusion criteria were an age >18, documented HIV test results, a gestational age between 32 and 36 weeks at enrollment, willingness to attend monthly visits with the child, and the ability to provide informed consent [24]. The second cohort (SMAC cohort) included 288 Malawian HEU infants born to pregnant HIV-infected women who received antiretroviral therapy (ART) for the prevention of breastfeeding transmission and follow-up until they were 24 months of age between 2008 and 2011. Maternal inclusion criteria were an age > 16, being naïve to antiretrovirals (with the exception of single-dose nevirapine), willingness to breastfeed infants up to 6 months of age, and having no main laboratory abnormalities or active tuberculosis [25].

For the present analyses, all infants in the two cohorts with available samples were selected.

We measured samples of ferritin, retinol-binding protein, vitamin D, C-reactive protein, and alpha1-acid glycoprotein extracted from these two cohorts of breastfed infants at 6 months of age. Inclusion of data in this analysis was based on sample availability.

### 2.2. Clinical Procedures

In the first cohort (GF cohort), infants were treated in the urban center of Blantyre and the two periurban sites of Chileka and Machinjiri. In the second cohort (SMAC cohort), infants were treated in the urban center of Blantyre (same center as above) and the periurban center of Lilongwe. In both cohorts, infant visits occurred monthly during the first year of life. Infant vaccinations and maternal antiretroviral treatment followed the Malawi National Guidelines [26,27]. In the most recent cohort, most HIV-positive women on ART were prescribed tenofovir, lamivudine, and efavirenz (76.4%) [24], while in the older cohort, women ART were prescribed either zidovudine, lamivudine, and nevirapine (46.9%) or stavudine, lamivudine, and nevirapine (53.1%) [25].

A PCR was performed in infants for HIV-DNA detection at 6 and 48 weeks, and all the infants included in this analysis were HIV-negative.

### 2.3. Sample Collection and Analysis

Infant blood was collected from peripheral veins during visits at 6 months. Serum or plasma samples were deidentified using pseudonomyzation, stored at –80 °C, and shipped in dry ice to the Laboratory of the National Center for Global Health of the Istituto Superiore di Sanità, where they were stored at −80 °C until processing.

RBP was used as an indicator of vitamin A status [17,28,29]. An automatized nephelometry (BN ProSpec^®^ System analyzer, Siemens Healthcare Diagnostics) was used to quantify plasma levels of ɑ₁-acid glycoprotein, retinol-binding protein, ferritin, and C-reactive protein (CRP2, sensitive) based on manufacturer’s instructions (BN ProSpec^®^ System Assay Protocols, Siemens Healthcare Diagnostics, Marburg, Germany). Plasma levels of vitamin D were quantified using the Euroimmun Medizinische Labordiagnostika AG (Lübeck, Germany, cod N. EQ 6411-9601) and the MyBioSource assay (San Diego, CA, USA, Catalog N. MBS580159) based on the manufacturer’s’ instructions.

Due to the known effect of inflammation on levels of ferritin and RBP [30], the levels of these biomarkers were corrected to adjust for the effect of inflammation by applying arithmetic correction factors (CF). Based on the measured levels of CRP (elevated if >5 mg/L) and AGP (elevated if > 1 g/L) [31,32,33], subjects’ inflammation statuses were grouped into the following four categories: incubation (elevated CRP only), early convalescence (elevated CRP and AGP), late convalescence (elevated AGP only), and reference (no elevated APP). The arithmetic conversion factors used for incubation, early convalescence, and late convalescence, which were derived from the BRINDA project, were 0.68, 0.38, and 0.65 for ferritin [32] and 1.22, 1.38, and 1.09 for RBP [33], respectively.

### 2.4. Definitions

Feeding was categorized as exclusive breastfeeding (infants who only received breast milk), mixed breastfeeding (infants who received breast milk supplemented with other foods or liquids, including traditional medicines and water), or formula feeding (infants who exclusively received replacement feeds and no breast milk). Mothers were encouraged to exclusively breastfeed infants during their first 6 months of life [34].

Length for age, weight for age, and weight for length Z-scores (LAZ, WAZ, WLZ, respectively) were calculated using the WHO Anthro Survey Analyzer, excluding values of less than −6 and greater than 6 as implausible [35]. We defined stunting as a LAZ of less than −2, being underweight as a WAZ of less than −2, and wasting as a WLZ of less than −2 [9,36].

Vitamin D status was categorized according to serum 25(OH)D concentrations, which were defined as severely deficient (<30 nmol/L), deficient (<50 nmol/L), insufficient (50–74 nmol/L), and sufficient (≥75 nmol/L) [14,37]. Vitamin A deficiency was defined based on RBP concentrations < 0.7 micromol/L [33], and iron deficiency was defined based on ferritin concentrations < 12 micrograms/L [32].

### 2.5. Statistical Analysis

The final sample size was not defined a priori and was based on all laboratory samples available for analysis. Data were summarized as proportions and means with standard deviations (SD) or medians, as well as interquartile (25–75 percentile) ranges. Stunting, being underweight, and wasting were evaluated at six and twelve months. Concentrations of biomarkers and other quantitative variables were compared via the T test or the Mann–Whitney U test. Categorical variables (including micronutrient deficiencies, being underweight, stunting, and wasting) were compared via either the chi-square test or the Fisher test (when expected cell frequencies were <5). Odds ratios (with 95% confidence intervals) were calculated using contingency tables. In all analyses, *p* values < 0.05 were considered significant, with no correction for multiple comparisons. All analyses were performed using SPSS software version 27 (IBM Corp, 2017, Armonk, NY, USA).

## 3. Results

The study included 88 infants born to 86 mothers (two sets of twins), of whom 63 infants were from the GF cohort and 25 were from the SMAC cohort; 14 infants were HUU (all in the GF cohort) and 74 HEU (49 in the GF cohort and 25 in the SMAC cohort). The general characteristics of mothers and infants are reported in Table 1. Within the more recent cohort (GF), no significant differences were found between HIV-positive and HIV-negative women. Considering only HIV-positive women, individuals from the earlier cohort (SMAC) had lower haemoglobin levels (10.0 vs. 11.0 g/dL) and worse clinical HIV disease stage than those from the more recent cohort (SMAC: 12% symptomatic HIV disease vs. 0% in the GF group). Similarly, the HIV-exposed and HIV-unexposed infants of the more recent cohort showed no differences related to birth weight, low birth weight, sex, month of weaning, and season of birth, while HEU infants of the earlier cohort, compared to HEU of the more recent cohort, were more commonly female and started mixed feeding at an earlier stage in life, with statistically non-significant trends for more frequent birth in winter and lower birth weight (Table 1).

The status of infants with respect to body weight and height, inflammatory indexes, and nutritional parameters is reported in Table 2. HEU and HUU infants from the more recent cohort did not differ in terms of body parameters, inflammatory indexes, and levels of RBP, ferritin, and vitamin D. HEU infants in the earlier cohort, compared to the HEU infants in the more recent cohort, had similar micronutrient levels, but significantly worse parameters for all body indexes (weight, height, weight for age, weight for length, and length for age), although rates of being underweight, stunting, and wasting were not significantly different between the two HEU groups (Table 2). Overall, almost half of the infants (48.9%) had levels of AGP or CRP that defined inflammation, and more than two-thirds of infants had ferritin deficiency (68.6%). Vitamin A deficiency was much less common (8.0%). None of the infants had severely deficient vitamin D levels, only one infant (1.2%) had vitamin D deficiency (HEU infant from the more recent cohort), and only four infants (4.8%, all GF HEU) had vitamin D insufficiency. None of the HUU infants had either vitamin D deficiency (<50 nmol/L) or insufficiency (<75 nmol/L).

Infant growth was analyzed by assessing changes in body parameters (weight and height gain, differences in BMI, BMI z-score, WAZ, LAZ and WLZ) between 6 and 12 months of age. The results of these analyses are reported in Table 3. Once again, no differences were found between HEU and HUU infants, while HEU infants in the earlier cohort, compared to their counterparts in the more recent cohort, had lower growth in weight and height (0.8 vs. 1.2 kg and 5.7 vs. 8.0 cm, respectively), which was accompanied by significantly worse changes in WAZ (−0.84 vs. −0.30) and LAZ (−0.84 vs. −0.07) (Table 3).

We then analyzed possible associations between micronutrient status and growth. No significant associations were found between RBP deficiency or vitamin D insufficiency at 6 months and subsequent growth between 6 and 12 months, while iron deficiency (ferritin levels <12 micrograms/L) was significantly associated with lower weight gain between 6 and 12 months (1.1 vs. 1.8 kg in infants with normal ferritin at 6 months), as well as significant decreases, compared to maintained or increased values in the other group, in both the BMI Z-score (−0.63 vs. +0.31) and the weight for age Z-score (−0.58 vs. + 0.12). (Table 3).

We, finally, assessed possible associations between micronutrient deficiencies and nutritional status, considering micronutrient levels at six months and the occurrence of stunting, being underweight, and wasting at either 6 or 12 months. The rates of micronutrient deficiency or insufficiency were not significantly different between infants with and without stunting and wasting and who were or were not underweight (Table 4).

## 4. Discussion

Using two cohorts of HIV-uninfected and breastfed Malawian infants, we evaluated several clinical, demographic, and biochemical variables, including micronutrient levels (ferritin, retinol-binding protein, vitamin D), markers of inflammation (C-reactive protein and alpha1-acid glycoprotein), and growth and nutritional status in the first year of life. The earlier cohort only included HIV-exposed infants, while the more recent cohort, which was separated from the first cohort by a ten-year temporal interval, included both HIV-exposed and HIV-unexposed infants. This method allowed us to evaluate and describe clinical, biochemical, and nutritional aspects not only according to sociodemographic and clinical factors, but also based on HIV exposure status and calendar time, thus providing potentially relevant information.

The findings related to the HIV-exposed cohorts indicate an overall improvement over time in the health of both mothers with HIV and their infants: HIV-infected mothers from the earlier cohort had significantly lower haemoglobin levels at third trimester of pregnancy (10.0 vs. 11.0 g/dL) and more frequently showed symptoms of HIV-related conditions (12.0% vs. 0%). Data regarding infants at birth showed the same trend: HEU infants from the earlier cohort had, when compared to their more recent counterparts, lower birthweight (3.2 vs. 3.4 kg), and they were more commonly below the threshold of low birthweight (<2500 g), i.e., 21.7% and 3.6%, respectively. These differences, although clinically relevant, were, however, not significant (*p*: 0.063 and 0.079, respectively) due to the limited sample size. The differences between the two HEU groups became significant at six months of age, when HEU infants from the earlier cohort had worse scores for all clinical anthropometric parameters evaluated, i.e., weight, height, BMI, and Z-scores for BMI; weight for age; length for age; and weight for length. These differences were maintained in the second semester of life, with significant worse growth indexes (differences in weight, height, weight for age, and length for age between six and twelve months) in children in the earlier cohort. Rates of being underweight, stunting, or wasting were, however, not significantly different between the two HEU groups. The analyses of biomarkers of inflammation and comparison of micronutrient levels also showed no significant differences between the HEU infants in the two cohorts.

Our findings confirm the progress achieved in recent years in improving maternal and infant health among women with HIV and their children [38,39,40,41,42]. Such advances can be attributed to several interventions implemented in Africa during the last two decades, which include wider dissemination of HIV programs [38], increased coverage of maternal antiretroviral treatment [43], integration of HIV services and other health services [40,42], involvement of community health workers [44], and introduction of infant vaccination for rotavirus and pneumococcus [45].

Our analysis of micronutrient status showed that the rates of micronutrient deficiencies, although similar in the subgroups analyzed, were markedly different for ferritin, RBP, and vitamin D. Ferritin deficiency was extremely common, affecting more than two thirds of infants at six months, with no significant differences based on HIV exposure or the temporal cohort; RBP deficiency was much less frequent, affecting only 8% of infants at six months, and, once again, no significant differences were observed between subgroups. Finally, vitamin D deficiency was observed in only one infant, and insufficiency was observed in less than 5% of infants at six months of age. No differences were observed between subgroups, though the numbers were small. No significant differences were observed based on infant HIV exposure and temporal cohort in the absolute plasma levels of the three micronutrients studied. Due to the potential effect of inflammation on ferritin and retinol-binding protein, the plasma concentrations of these two proteins were adjusted according to the inflammatory status defined based on levels of two acute-phase proteins that were concomitantly measured [31,32,33].

The present data contribute new information regarding the micronutrient status of African infants. A high rate of iron deficiency in childhood has been described in several African countries, being particularly high in infants and pre-school children, before declining as the child’s age increases [46,47]. Our data, which show a 68.6% rate of iron deficiency according to ferritin levels in six-month-old infants, with no significant differences between the HIV-exposed and HIV unexposed groups, confirm the high prevalence of this condition at the age of weaning, and they reinforce the need to implement nutrient supplementations, which have been proven effective in ameliorating iron status and anemia [46,48].

Our analysis of vitamin A status (assessed through concentrations of retinol-binding protein) showed an overall prevalence of vitamin A deficiency of 8% at six months of age, with no significant differences between subgroups. This rate is fully consistent with the 8.9% rate observed in Ugandan children aged 6–59 months old who were evaluated in 2016 [17], but below the prevalence range of 14–42% reported in a systematic review based on data from four African countries collected between 2005 and 2016 [49]. The differences in the geographical contexts and populations studied make difficult to draw conclusions, though the lower rates observed in more recent years might indicate an improvement in population health over time.

With respect to vitamin D status, the information provided contributes to the open issue of prevalence of vitamin D deficiency in Africa, which is characterized by large variability among countries based on the reported prevalence of this condition [37]. The low rates of deficiency and insufficiency observed in our study in six-month-old African infants provide some reassurance, confirming that vitamin D deficiency is less common in infants this age or older than in neonates or 6–10-week-old infants [50,51,52,53,54], with no apparent negative effect related to HIV exposure. Vitamin D supplementation was not provided to infants in this study. Infant age, feeding, and solar exposure are likely to play a major role in final vitamin D status, because breast milk is considered to be a poor source of vitamin D, and the levels of vitamin D increase with age during the first year of life, being comparable to those of adults by 12 months of age [54]. Children from Sub-Saharan Africa also have generally higher vitamin D levels than those from Western populations [55,56].

We, finally, assessed infant growth between six and twelve months in the population studied. Infant growth between six and twelve months was similar for all parameters in HEU and HUU infants, but lower for weight, height, weight for age, and length for age in HEU infants of the earlier cohort than in their more recent counterparts. This finding indicates that the gap that we observed between the two HEU cohorts at six months, which is characterized by better infant anthropometric measures in the more recent population, is maintained until twelve months of age.

The analysis of the possible associations between growth and micronutrient status showed a significantly worse rate of growth between six and twelve months in infants with ferritin deficiency. This result indirectly confirms previous observations of significant associations between iron deficiency and stunting [57], and it reinforces the rationale for administering iron supplementations to infants in order to prevent not only occurrence of anemia, but also suboptimal growth and stunting.

In the entire population, the markers of malnutrition had different prevalence rates: at six months, stunting (25.0%) was much more common than being underweight (2.7%) or wasting (2.8%), suggesting chronic undernutrition. These rates were not significantly different based on HIV exposure status or temporal cohort, though the numbers of cases of being underweight and wasting were low. We were also unable to identify associations between stunting, being underweight, and wasting and micronutrient deficiency (ferritin, RBP) or insufficiency (vitamin D). The data do not confirm other findings of higher prevalence of stunting in HEU than HUU infants [58], but they indicate that stunting, although decreasing, still affected a significant proportion of pre-school infants in Africa, irrespective of maternal HIV status, possibly due to socioeconomic and demographic factors [57,59,60,61,62,63]. Overall, the findings suggest that the current population of HIV-exposed infants does not need specific interventions regarding the nutritional aspects evaluated, because the deficiencies observed involved similarly HIV-exposed and HIV-unexposed infants.

The main limitation of the present study is represented by its limited sample size. Inclusion criteria were essentially based on the availability of samples from the two cohorts. We had already evaluated several immunological parameters in the infants of the two cohorts [64,65,66,67], and we used all residual samples to perform the present laboratory analyses, examining the clinical correlates of the laboratory findings. This method translated into an intensive evaluation of a small sample, which had limited statistical power for some analyses. Some associations that approached significance could be explored via future larger studies. Despite the limited sample size, the study was able to detect significant differences of clinical relevance, such as the significantly better clinical status in the more recent cohort of women with HIV and their infants. Almost all infant anthropometric indexes and growth measures were significantly better in the more recent cohort, supporting the hypothesis that recent programs have significantly improved the health status of HIV-exposed infants. The gradual reduction in the gap between this population and the general population of HIV-unexposed infants is also supported by the absence of significant differences between the two contemporary HEU and HUU groups for all variables considered, which included a wide range of clinical and laboratory parameters. An additional possible limitation of our study is that our intensive evaluation required a high number of comparisons between groups, and the high number of tests performed may have increased the possibility of finding falsely positive associations.

Our study also identified some areas of relevance for potential interventions, such as the maintained high rates of stunting in both HIV-exposed and HIV-unexposed infants, as well as the significant association between ferritin deficiency and worse subsequent growth, which suggest that iron deficiency might indicate infants at risk of impaired growth who could benefit from appropriate nutritional supplements. All of the above findings can be of interest in terms of both setting future research and public health agendas.

## Figures and Tables

**Table 1:** Population.

**Mothers (N = 86)**	**All Women**	**GF Cohort, All**	**GF Cohort,** **HIV−**	**GF Cohort,** **HIV+**	**SMAC Cohort** **(all HIV+)**	***p* Value**	***p* Value**
	**% (n/N)**	**% (n/N)**	**% (n/N)**	**% (n/N)**	**% (n/N)**	**GF HIV+ vs.** **GF HIV−**	**GF HIV+ vs. SMAC HIV+**
Education level							
None/primary	60.5 (52/86)	55.7 (34/61)	61.5 (8/13)	54.2 (26/48)	72.0 (18/25)	0.635	0.140
Secondary/above secondary	39.5 (34/86)	44.3 (27/61)	38.5 (5/13)	45.8 (22/48)	28.0 (7/25)		
Working status							
Housewife/none	69.8 (60/86)	68.9 (42/61)	84.6 (11/13)	64.6 (31/48)	72.0 (18/25)	0.311	0.522
Trader/other job	30.2 (26/86)	31.1 (19/61)	15.4 (2/13)	35.4 (17/48)	28.0 (7/25)		
Place of delivery							
Health center	61.6 (53/86)	65.6 (40/61)	84.6 (11/13)	60.4 (29/48)	48.0 (13/25)	0.187	0.490
Hospital	38.4 (33/86)	34.4 (21/61)	15.4 (2/13)	39.6 (19/48)	52.0 (12/25)		
Electricity in household	37.6 (32/85)	39.3 (24/61)	38.5 (5/13)	39.6 (19/48)	33.3 (8/24)	0.941	0.606
WHO HIV stage I (asymptomatic)	96.5 (83/86)	100.0 (61/61)	100.0 (13/13)	100.0 (48/48)	88.0 (22/25)	-	0.037
Underweight (BMI < 18.5) ^†^	3/80 (3.8)	2/56 (3.6)	0/12 (0)	2/44 (4.5)	1/24 (4.2)	1.000	1.000
	**Median (IQR)**	**Median (IQR)**	**Median (IQR)**	**Median (IQR)**	**Median (IQR)**	**GF HIV+ vs. GF HIV−**	**GF HIV+ vs. SMAC HIV+**
Age (years) (n: 86)	29.5 (23.7–33.0)	30 (24.0–33.5)	30.0 (25.5–33.5)	30.0 (23.0–33.7)	27.0 (22.0–32.0)	0.812	0.303
Number of other living children (n: 86)	2 (1–3)	2 (1–3)	2 (1.5–3)	2 (1–3)	2 (0.5–3)	0.396	0.976
Haemoglobin (g/dL, 3rd trimester) (n: 60)	10.7 (9.6–11.5)	11.0 (10.5–11.9)	13.0 (13.0–13.0)	11.0 (10.5–11.9)	10.0 (9.0–10.0)	0.056	<0.001
Body mass index (kg/m^2^) (n: 80) ^†^	22.8 (20.9–25.6)	23.0 (21.3–26.4)	24.7 (22.1–26.9)	22.8 (20.7–26.0)	22.3 (20.4–23.9)	0.194	0.464
**Infants (N = 88)**	**All Infants**	**GF Cohort, All**	**GF Cohort, HUU**	**GF Cohort, HEU**	**SMAC Cohort, HEU**	***p* Value**	***p* Value**
	**Median (IQR)**	**Median (IQR)**	**Median (IQR)**	**Median (IQR)**	**Median (IQR)**	**GF HEU vs. GF HUU**	**GF HEU vs. SMAC HEU**
Birth weight (Kg) (n: 58)	3.30 (3.00–3.73)	3.4 (3.0–3.9)	3.4 (2.7–3.7)	3.4 (3.02–3.97)	3.20 (2.98–3.60)	0.558	0.063
Month of the start of mixed feeding (n: 88)	6 (6–7)	6 (6–7)	6 (6.0–6.25)	6 (6–7)	6 (6–6)	0.256	0.013
	**% (n/N)**	**% (n/N)**	**% (n/N)**	**% (n/N)**	**% (n/N)**	**GF HEU vs. GF HUU**	**GF HEU vs. SMAC HEU**
Gender (n: 88)							
Female	45.5 (40/88)	39.7 (25/63)	57.1 (8/14)	34.7 (17/49)	60.0 (15/25)	0.130	0.038
Male	54.5 (48/88)	60.3 (38/63)	42.9 (6/14)	65.3 (32/49)	40.0 (10/25)		
Born in winter (n: 88)	28.4 (25/88)	34.9 (22/63)	42.9 (6/14)	32.7 (16/49)	12.0 (3/25)	0.480	0.090
Low birth weight (<2500 g) (n: 58)	12.1 (7/58)	5.7 (2/35)	14.3 (1/7)	3.6 (1/28)	21.7 (5/23)	0.365	0.079

IQR: interquartile range (between 25 and 75 percentile); ^†^ measured one month postpartum.

**Table 2:** Infant status at 6 months: weight/height parameters, micronutrients, inflammation, and haemoglobin.

**N: GF 63 (HUU 14, HEU 49);** **SMAC 25 (HEU)**	**All Infants**	**GF Cohort, All**	**GF Cohort, HUU**	**GF Cohort, HEU**	**SMAC Cohort, HEU**	***p* Value**	***p* Value**
	**Median (IQR)**	**Median (IQR)**	**Median (IQR)**	**Median (IQR)**	**Median (IQR)**	**GF HEU vs.** **GF HUU**	**GF HEU vs.** **SMAC HEU**
Weight, Kg (n: 75)	7.5 (6.9–8.0)	7.75 (7.0–8.5)	7.7 (6.9–8.9)	7.8 (7.0–8.4)	7.1 (6.4–7.5)	0.992	0.002
Height, cm (n: 72)	65.0 (63.0–66.0)	66.0 (63–67)	65.0 (62.5–66.0)	66.0 (64.0–67.2)	64 (61.5–65.7)	0.198	0.040
Body mass index, Kg/m^2^ (n: 72)	17.6 (16.8–19.4)	18.4 (17.1–20.3)	18.9 (17.0–21.0)	18.3 (17.1–19.7)	17.0 (16.0–17.7)	0.399	0.005
BMI Z-score (n: 72)	0.34 (−0.37–1.38)	0.79 (−0.04–1.95)	1.23 (−0.07–2.32)	0.75 (−0.06–1.56)	−0.25 (−0.62–0.39)	0.364	0.006
Weight for age Z-score (n: 75)	−0.26 (−1.05–0.63)	−0.09 (−0.97–0.76)	0.35 (−1.16–1.19)	−0.12 (−0.91–0.74)	−0.78 (−1.35–0.25)	0.693	0.006
Length for age Z-score (n: 72)	−1.11 (−1.99–0.09)	−1.08 (−2.03–0.05)	−1.74 (−2.41–0.04)	−1.06 (−2.01–0.01)	−1.4 (−1.88–0.69)	0.411	0.027
Weight for length Z-score (n: 72)	0.46 (−0.16–1.45)	1.00 (0.05–2.03)	1.31 (0.12–2.34)	0.94 (0.03–1.91)	−0.12 (−0.42–0.56)	0.376	0.008
Haemoglobin, g/dL (n: 21)	10.0 (10.0–11.0)	-	-	-	10.0 (10.0–11.0)	-	-
Alpha-glycoprotein, g/L (n: 88)	0.98 (0.79–1.27)	0.92 (0.76–1.26)	1.11 (0.84–1.36)	0.87 (0.73–1.21)	1.06 (0.84–1.31)	0.175	0.151
C-reactive protein, mg/L (n: 88)	0.92 (0.24–4.88)	0.72 (0.24–4.77)	1.52 (0.42–7.53)	0.48 (0.22–3.70)	1.15 (0.21–6.60)	0.164	0.369
Ferritin, μg/L (n: 86)	6.60 (2.95–16.51)	7.02 (3.17–17.49)	11.70 (3.84–22.69)	7.00 (3.00–15.24)	5.27 (2.38–9.63)	0.372	0.203
Retinol-binding protein, μmol/L (n: 88)	1.37 (1.02–1.82)	1.35 (0.97–1.72)	1.30 (0.96–1.79)	1.38 (0.98–1.73)	1.41 (1.24–2.29)	0.766	0.170
Vitamin D, nmol/L (n: 84)	134 (115–170)	129 (112–168)	136 (115–175)	128 (111–162)	152 (119–175)	0.395	0.087
	**All Infants**	**GF Cohort, all**	**GF Cohort, HUU**	**GF Cohort, HEU**	**SMAC Cohort, HEU**	***p* Value**	***p* Value**
	**% (n/N)**	**% (n/N)**	**% (n/N)**	**% (n/N)**	**% (n/N)**	**GF HEU vs. GF HUU**	**GF HEU vs. SMAC HEU**
Inflammation status (n: 88)							
No inflammation	51.1 (45/88)	55.6 (35/63)	42.9 (6/14)	59.2 (29/49)	40.0 (10/25)	0.278	0.118
Incubation	1.1 (1/88)	1.6 (1/63)	0 (0/14)	2.0 (1/49)	0 (0/25)	1.000	1.000
Early convalescence	22.7 (20/88)	20.6 (13/63)	28.6 (4/14)	18.4 (9/49)	28.0 (7/25)	0.461	0.341
Late convalescence	25.0 (22/88)	22.2 (14/63)	28.6 (4/14)	20.4 (10/49)	32.0 (8/25)	0.492	0.272
Ferritin deficiency (<12 μg/L) (n: 86)	68.6 (59/86)	63.9 (39/61)	50.0 (7/14)	68.1 (32/47)	80.0 (20/25)	0.216	0.263
Vitamin A deficiency (RBP < 0.7 μmol/L) (n: 88)	8.0 (7/88)	7.9 (5/63)	14.3 (2/14)	6.1 (3/49)	8.0 (2/25)	0.307	1.000
Vitamin D deficiency (<50 nmol/L) (n: 84)	1.2 (1/84)	1.7 (1/59)	0 (0/13)	2.2 (1/45)	0 (0/25)	1.000	1.000
Vitamin D insufficiency (<75 nmol/L) (n: 84)	4.8 (4/84)	6.8 (4/59)	0 (0/13)	8.7 (4/46)	0 (0/25)	0.566	0.290
Underweight (WAZ < −2) (n: 75)	2.7 (2/75)	1.9 (1/54)	0 (0/13)	2.4 (1/41)	4.8 (1/21)	1.000	1.000
Stunting (LAZ < −2) (n: 72)	25.0 (18/72)	27.5 (14/51)	30.8 (4/13)	26.3 (10/38)	19.0 (4/21)	0.734	0.751
Wasting (WLZ < −2) (n: 72)	2.8 (2/72)	3.9 (2/51)	0 (0/13)	5.3 (2/38)	0 (0/21)	1.000	0.534

Ferritin and RBP adjusted for inflammation [31,32,33]; IQR: interquartile range (between 25 and 75 percentile); RBP: retinol-binding protein; WAZ: weight for age Z-score; LAZ: length for age Z-score; WLZ: weight for length Z-score.

**Table 3:** Infant growth: changes between 6 and 12 months of age in body parameters.

	**All Infants**			**GF Cohort**			**SMAC Cohort**		
**N: GF 40 (HUU 9, HEU 31);** **SMAC 16 (HEU)**		**GF Cohort, All**		**GF Cohort, HUU**	**GF Cohort, HEU**		**SMAC Cohort, HEU**	***p* Value**	***p* Value**
	**Median (IQR)**	**Median (IQR)**		**Median (IQR)**	**Median (IQR)**		**Median (IQR)**	**GF HEU vs. GF HUU**	**GF HEU vs. SMAC HEU**
Weight, Kg (n: 57)	1.2 (0.7–1.8)	1.3 (0.8–1.9)		1.4 (0.9–1.7)	1.2 (0.8–2.0)		0.8 (0.3–1.1)	0.963	0.013
Height, cm (n: 55)	7.0 (6.0–9.0)	7.5 (7.0–9.0)		7.0 (6.5–10.5)	8.0 (7.0–9.0)		5.7 (4.6–8.5)	0.919	0.015
Body mass index, Kg/m^2^ (n: 54)	−1.33 (−2.20–0.17)	−1.26 (−2.55–0.05)		−1.37 (−3.12–0.19)	−1.15 (−2.50–0.06)		−1.35 (−1.87–0.22)	0.711	0.906
BMI Z-score (n: 54)	−0.39 (−1.08–0.34)	−0.32 (−1.20–0.52)		−0.35 (−1.70–0.57)	−0.29 (−1.12–0.56)		−0.66 (−0.99–0.21)	0.589	0.553
Weight for age Z-score (n: 56)	−0.46 (−0.92–0.13)	−0.21 (−0.76–0.16)		0.09 (−0.73–0.12)	−0.30 (0.80–0.18)		−0.84 (−1.27–0.57)	0.949	0.014
Length for age Z-score (n: 53)	−0.18 (−0.88–0.56)	−0.08 (−0.53–0.60)		−0.08 (−0.88–1.14)	−0.07 (−0.50–0.61)		−0.84 (−1.41–0.24)	0.931	0.006
Weight for length Z-score (n: 54)	−0.86 (−1.47–0.03)	−0.77 (−1.48–0.18)		−0.74 (−2.08–0.20)	−0.81 (−1.43–0.20)		−0.96 (−1.40–0.23)	0.613	0.499
	**Ferritin Status**			**RBP Status**			**Vitamin D Status**		
	**Normal**	**Deficient**		**Normal**	**Deficient**		**Normal**	**Insufficient**	
	**Median (IQR)**	**Median (IQR)**	***p* Value**	**Median (IQR)**	**Median (IQR)**	***p* Value**	**Median (IQR)**	**Median (IQR)**	***p* Value**
Weight, Kg (n: 56)	1.8 (0.8–2.3)	1.1 (0.7–1.4)	0.016	1.1 (0.7–1.85)	1.5 (0.7–1.9)	0.466	1.1 (0.7–1.9)	1.2 (0.6–1.4)	0.861
Height, cm (n: 53)	8.0 (5.5–9.5)	7.0 (6.0–8.7)	0.584	7.0 (6.0–9.0)	7.0 (5.0–7.0)	0.196	7.0 (5.6–8.7)	8.0 (7.0–n.c.)	0.251
Body mass index, Kg/m^2^ (n: 53)	−0.19 (−2.49–1.00)	−1.48 (−2.39–0.80)	0.067	−1.37 (−2.50–0.19)	−0.76 (−1.31–0.75)	0.139	−1.33 (−1.99–0.01)	−2.69 (−3.75–n.c.)	0.164
BMI Z-score (n: 53)	0.31 (−1.20–0.94)	−0.63 (−1.08–0.08)	0.045	−0.55 (−1.12–0.28)	0.02 (−0.62–0.89)	0.217	−0.39 (−1.05–0.47)	−1.77 (−1.80–n.c.)	0.190
Weight for age Z-score (n: 55)	0.12 (−0.80–0.55)	−0.58 (−0.96–0.21)	0.017	−0.54 (−0.94–0.14)	0.11 (−0.80–0.14)	0.520	−0.48 (−0.94–0.13)	−0.52 (−0.91–0.38)	0.569
Length for age Z-score (n: 52)	−0.08 (−0.88–0.68)	−0.18 (−0.92–0.38)	0.668	−0.14 (−0.78–0.61)	−0.85 (−0.92–0.16)	0.225	−0.29 (−0.92–0.24)	0.57 (−0.19–n.c.)	0.161
Weight for length Z-score (n: 53)	−0.22 (−1.46–0.62)	−0.98 (−1.49–0.25)	0.060	−0.92 (−1.49–0.09)	−0.46 (−1.03–0.64)	0.206	−0.86 (−1.43–0.15)	−1.59 (−2.33–n.c.)	0.304

**Table 4:** Analysis of possible associations between micronutrient deficiency (ferritin, vitamin A) or insufficiency (vitamin D) and nutritional status at 6 and 12 months.

	Underweight at Any Time (6 or 12 Months)			Stunting at Any Time (6 or 12 Months)			Wasting at Any Time (6 or 12 Months)		
	Yes	No		Yes	No		Yes	No	
Micronutrient Status at 6 Months	% (n/N)	% (n/N)	*p* Value	% (n/N)	% (n/N)	*p* Value	% (n/N)	% (n/N)	*p* Value
Ferritin deficiency (n: 83)									
Yes	80.0 (8/10)	67.1 (49/73)	0.417	72.0 (18/25)	67.2 (39/58)	0.668	77.8 (7/9)	67.6 (50/74)	0.713
No	20.0 (2/10)	32.9 (24/73)		28.0 (7/25)	32.8 (19/58)		22.2 (2/9)	32.4 (24/74)	
Vitamin A deficiency (n: 85)									
Yes	10.0 (1/10)	8.0 (6/75)	1.000	11.5 (3/26)	6.8 (4/59)	0.670	0 (0/9)	9.2 (7/76)	1.000
No	90.0 (9/10)	92.0 (69/75)		88.5 (23/26)	93.2 (55/59)		100.0 (9/9)	90.8 (69/76)	
Vitamin D insufficiency (n: 81)									
Yes	0 (0/9)	5.6 (4/72)	1.000	4.3 (1/23)	5.2 (3/58)	1.000	12.5 (1/8)	4.1 (3/73)	0.346
No	100.0 (9/9)	94.4 (68/72)		95.7 (22/23)	94.8 (55/58)		87.5 (7/8)	95.9 (70/73)	

## Data Availability

The datasets used and analyzed during the current study are available from the corresponding author on reasonable request.

## References

[1] Perkins J.M., Kim R., Krishna A., McGovern M., Aguayo V.M., Subramanian S.V. (2017). Understanding the association between stunting and child development in low- and middle-income countries: Next steps for research and intervention. Soc. Sci. Med..

[2] Soekatri M.Y.E., Sandjaja S., Syauqy A. (2020). Stunting was associated with reported morbidity, parental education and socioeconomic status in 0.5–12-year-old Indonesian children. Int. J. Environ. Res. Public Health.

[3] Victora C.G., de Onis M., Hallal P.C., Blossner M., Shrimpton R.C. (2020). Worldwide timing of growth faltering: Revisiting implications for interventions. Pediatrics.

[4] Dewey K.G., Begum K. (2011). Long-term consequences of stunting in early life. Matern. Child Nutr..

[5] Kinyoki D.K., Berkley J.A., Moloney G.M., Kandala N.B., Noor A.M. (2015). Predictors of the risk of malnutrition among children under the age of 5 years in Somalia. Public Health Nutr..

[6] World Health Organization, United Nations Children’s Fund (UNICEF), World Bank (2021). Levels and Trends in Child Malnutrition: UNICEF/WHO/The World Bank Group Joint Child Malnutrition Estimates: Key Findings of the 2021 Edition.

[7] WHO (2013). Guideline: Updates on the Management of Severe Acute Malnutrition in Infants and Children.

[8] Evans C., Jones C.E., Prendergast A.J. (2016). HIV-exposed, uninfected infants: New global challenges in the era of paediatric HIV elimination. Lancet Infect. Dis..

[9] Yeganeh N., Watts D.H., Xu J., Kerin T., Joao E.C., Pilotto J.H., Theron G., Gray G., Santos B., Fonseca R. (2018). Infectious Morbidity, Mortality and Nutrition in HIV-exposed, Uninfected, Formula-fed Infants: Results From the HPTN 040/PACTG 1043 Trial. Pediatr. Infect. Dis. J..

[10] le Roux S.M., Abrams E.J., Donald K.A., Brittain K., Phillips T.K., Zerbe A., le Roux D.M., Kroon M., Myer L. (2020). Infectious morbidity of breastfed, HIV-exposed uninfected infants under conditions of universal antiretroviral therapy in South Africa: A prospective cohort study. Lancet Child Adolesc. Health.

[11] Anderson K., Kalk E., Madlala H.P., Nyemba D.C., Kassanjee R., Jacob N., Slogrove A., Smith M., Eley B.S., Cotton M.F. (2021). Increased infectious-cause hospitalization among infants who are HIV-exposed uninfected compared with HIV-unexposed. AIDS.

[12] Cusick S.E., Opoka R.O., Lund T.C., John C.C., Polgreen L.E. (2014). Vitamin D insufficiency is common in Ugandan children and is associated with severe malaria. PLoS ONE.

[13] Sudfeld C.R., Duggan C., Aboud S., Kupka R., Manji K.P., Kisenge R., Fawzi W.W. (2015). Vitamin D status is associated with mortality, morbidity, and growth failure among a prospective cohort of HIV-infected and HIV-exposed Tanzanian infants. J. Nutr..

[14] Aibana O., Huang C.C., Aboud S., Arnedo-Pena A., Becerra M.C., Bellido-Blasco J.B., Bhosale R., Calderon R., Chiang S., Contreras C. (2019). Vitamin D status and risk of incident tuberculosis disease: A nested case-control study, systematic review, and individual-participant data meta-analysis. PLoS Med..

[15] Bearden A., Van Winden K., Frederick T., Kono N., Operskalski E., Pandian R., Barton L., Stek A., Kovacs A. (2020). Low maternal vitamin D is associated with increased risk of congenital and peri/postnatal transmission of Cytomegalovirus in women with HIV. PLoS ONE..

[16] Akeredolu F.D., Akuse R.M., Mado S.M., Yusuf R. (2021). Relationship Between Serum Vitamin D Levels and Acute Pneumonia in Children Aged 1-59 Months in Nigeria. J. Trop. Pediatr..

[17] Ssentongo P., Ba D.M., Ssentongo A.E., Fronterre C., Whalen A., Yang Y., Ericson J.E., Chinchilli V.M. (2020). Association of vitamin A deficiency with early childhood stunting in Uganda: A population-based cross-sectional study. PLoS ONE.

[18] Grant F.K., Wanjala R., Low J., Levin C., Cole D.C., Okuku H.S., Ackatia-Armah R., Girard A.W. (2022). Association between infection and nutritional status among infants in a cohort study of vitamin A in western Kenya. Front. Nutr..

[19] Imdad A., Mayo-Wilson E., Haykal M.R., Regan A., Sidhu J., Smith A., Bhutta Z.A. (2022). Vitamin A supplementation for preventing morbidity and mortality in children from six months to five years of age. Cochrane Database Syst. Rev..

[20] Scott S.P., Chen-Edinboro L.P., Caulfield L.E., Murray-Kolb L.E. (2014). The impact of anemia on child mortality: An updated review. Nutrients.

[21] Liotta G., Marazzi M.C., Mothibi K.E., Zimba I., Amangoua E.E., Bonje E.K., Bossiky N.B., Robinson P.A., Scarcella P., Musokotwane K. (2015). Elimination of mother-to-child transmission of HIV infection: The drug resource enhancement against AIDS and malnutrition model. Int. J. Environ. Res. Public Health.

[22] Floridia M., Ciccacci F., Andreotti M., Hassane A., Sidumo Z., Magid N.A., Sotomane H., David M., Mutemba E., Cebola J. (2017). Tuberculosis case finding with combined rapid point-of-care assays (Xpert MTB/RIF and determine TB LAM) in HIV-positive individuals starting antiretroviral therapy in Mozambique. Clin. Infect. Dis..

[23] Ciccacci F., Tolno V.T., Doro Altan A.M., Liotta G., Orlando S., Mancinelli S., Palombi L., Marazzi M.C. (2019). Noncommunicable diseases burden and risk factors in a cohort of HIV+ elderly patients in Malawi. AIDS Res. Hum. Retroviruses.

[24] Floridia M., Orlando S., Andreotti M., Mphwere R., Kavalo T., Ciccacci F., Scarcella P., Marazzi M.C., Giuliano M. (2023). A 12-month prospective study of HIV-infected and HIV-uninfected women and their infants in Malawi: Comparative Analysis of Clinical Events and Infant Growth. Am. J. Trop. Med. Hyg..

[25] Giuliano M., Andreotti M., Liotta G., Jere H., Sagno J.B., Maulidi M., Mancinelli S., Buonomo E., Scarcella P., Pirillo M.F. (2013). Maternal antiretroviral therapy for the prevention of mother-to-child transmission of HIV in Malawi: Maternal and infant outcomes two years after delivery. PLoS ONE.

[26] National Immunization Schedule: Malawi Recommended Routine Immunization. http://www.vacfa.uct.ac.za/sites/default/files/image_tool/images/210/Immunization_Schedules/Malawi.pdf.

[27] Ministry of Health and Population, Malawi 4th Edition of the Malawi Guidelines for Clinical Management of HIV in Children and Adults. https://differentiatedservicedelivery.org/Portals/0/adam/Content/xc8bFLkQfECqbTEpxM8C9Q/File/Malawi%20Clinical%20HIV%20Guidelines%202019%20Addendumversion%208.1.pdf.

[28] Sandalinas F., Filteau S., Joy E.J.M., Segovia de la Revilla L., MacDougall A., Hopkins H. (2022). Measuring the impact of malaria infection on indicators of iron and vitamin A status: A systematic literature review and meta-analysis. Br. J. Nutr..

[29] Namaste S.M.L., Ou J., Williams A.M., Young M.F., Yu E.X., Suchdev P.S. (2019). Adjusting iron and vitamin A status in settings of inflammation: A sensitivity analysis of the Biomarkers Reflecting Inflammation and Nutritional Determinants of Anemia (BRINDA) approach. Am. J. Clin. Nutr..

[30] Tomkins A. (2003). Assessing micronutrient status in the presence of inflammation. J. Nutr..

[31] Thurnham D.I., McCabe L.D., Haldar S., Wieringa F.T., Northrop-Clewes C.A., McCabe G.P. (2010). Adjusting plasma ferritin concentrations to remove the effects of subclinical inflammation in the assessment of iron deficiency: A meta-analysis. Am. J. Clin. Nutr..

[32] Namaste S.M., Rohner F., Huang J., Bhushan N.L., Flores-Ayala R., Kupka R., Mei Z., Rawt R., Williams A.M., Raiten D.J. (2017). Adjusting ferritin concentrations for inflammation: Biomarkers Reflecting Inflammation and Nutritional Determinants of Anemia (BRINDA) project. Am. J. Clin. Nutr..

[33] Larson L.M., Namaste S.M., Williams A.M., Engle-Stone R., Addo O.Y., Suchdev P.S., Wirth J.P., Temple V., Serdula M., Northrop-Clewes C.A. (2017). Adjusting retinol-binding protein concentrations for inflammation: Biomarkers Reflecting Inflammation and Nutritional Determinants of Anemia (BRINDA) project. Am. J. Clin. Nutr..

[34] World Health Organisation (2016). Guideline: Updates on HIV and Infant Feeding.

[35] WHO WHO Anthro Survey Analyzer and Other Tools. https://www.who.int/tools/child-growth-standards/software.

[36] Aizire J., Sikorskii A., Ogwang L.W., Kawalazira R., Mutebe A., Familiar-Lopez I., MacPherson M., Taha T., Boivin M.J., Glenn Fowler M. (2020). Decreased growth among antiretroviral drug and HIV-exposed uninfected versus unexposed children in Malawi and Uganda. AIDS.

[37] Mogire R.M., Mutua A., Kimita W., Kamau A., Bejon P., Pettifor J.M., Adeyemo A., Williams T.N., Atkinson S.A. (2020). Prevalence of vitamin D deficiency in Africa: A systematic review and meta-analysis. Lancet Glob. Health.

[38] Kruk M.E., Jakubowski A., Rabkin M., Kimanga D.O., Kundu F., Lim T., Lumumba V., Oluoch T., Robinson K.A., El-Sadr W. (2015). Association between HIV programs and quality of maternal health inputs and processes in Kenya. Am. J. Public Health.

[39] Goga A., Feucht U., Zar H.J., Vanker A., Wiysonge C.S., McKerrow N., Wright C.Y., Loveday M., Odendaal W., Ramokolo V. (2019). Neonatal, infant and child health in South Africa: Reflecting on the past towards a better future. S. Afr. Med. J..

[40] Bulstra C.A., Hontelez J.A.C., Otto M., Stepanova A., Lamontagne E., Yakusik A., El-Sadr W.M., Apollo T., Rabkin M., UNAIDS Expert Group on Integration (2021). Integrating HIV services and other health services: A systematic review and meta-analysis. PLoS Med..

[41] Achoki T., Sartorius B., Watkins D., Glenn S.D., Kengne A.P., Oni T., Wiysonge C.S., Walker A., Adetokunboh O.O., Kayode Babalola T. (2022). Health trends, inequalities and opportunities in South Africa’s provinces, 1990-2019: Findings from the Global Burden of Disease 2019 Study. J. Epidemiol. Community Health.

[42] Fonzo M., Zuanna T.D., Amoruso I., Resti C., Tsegaye A., Azzimonti G., Sgorbissa B., Centomo M., Ferretti S., Manenti F. (2023). The HIV paradox: Perinatal mortality is lower in HIV-positive mothers. A field case-control study in Ethiopia. Int. J. Gynaecol. Obstet..

[43] Sutcliffe C.G., Moyo N., Hamahuwa M., Mutanga J.N., van Dijk J.H., Hamangaba F., Schue J.L., Thuma P.E., Moss W.J. (2023). The Evolving Pediatric HIV Epidemic in Rural Southern Zambia: The Beneficial Impact of Advances in Prevention and Treatment at a District Hospital From 2007 to 2019. Pediatr. Infect. Dis. J..

[44] le Roux K.W., Almirol E., Rezvan P.H., le Roux I.M., Mbewu N., Dippenaar E., Stansert-Katzen L., Baker V., Tomlinson M., Rotheram-Borus M.J. (2020). Community health workers impact on maternal and child health outcomes in rural South Africa—A non-randomized two-group comparison study. BMC Public Health.

[45] Ajibola G., Bennett K., Powis K.M., Hughes M.D., Leidner J., Kgole S., Batlang O., Mmalane M., Makhema J., Lockman S. (2020). Decreased diarrheal and respiratory disease in HIV exposed uninfected children following vaccination with rotavirus and pneumococcal conjugate vaccines. PLoS ONE.

[46] Lemoine A., Tounian P. (2020). Childhood anemia and iron deficiency in sub-Saharan Africa—Risk factors and prevention: A review. Arch. Pediatr..

[47] Wegmüller R., Bentil H., Wirth J.P., Petry N., Tanumihardjo S.A., Allen L., Williams T.N., Selenje L., Mahama A., Amoaful E. (2020). Anemia, micronutrient deficiencies, malaria, hemoglobinopathies and malnutrition in young children and non-pregnant women in Ghana: Findings from a national survey. PLoS ONE.

[48] Stewart C.P., Fernald L.C.H., Weber A.M., Arnold C., Galasso E. (2020). Lipid-Based Nutrient Supplementation Reduces Child Anemia and Increases Micronutrient Status in Madagascar: A Multiarm Cluster-Randomized Controlled Trial. J. Nutr..

[49] Harika R., Faber M., Samuel F., Mulugeta A., Kimiywe J., Eilander A. (2017). Are Low Intakes and Deficiencies in Iron, Vitamin A, Zinc, and Iodine of Public Health Concern in Ethiopian, Kenyan, Nigerian, and South African Children and Adolescents?. Food Nutr. Bull..

[50] Bhimji K.M., Naburi H., Aboud S., Manji K. (2018). Vitamin D Status and Associated Factors in Neonates in a Resource Constrained Setting. Int. J. Pediatr..

[51] Tindall A.M., Schall J.I., Seme B., Ratshaa B., Tolle M., Nnyepi M.S., Mahzani L., Rutstein R.M., Steenhoff A.P., Stallings V.A. (2020). Vitamin D status, nutrition and growth in HIV-infected mothers and HIV-exposed infants and children in Botswana. PLoS ONE.

[52] Said N.A., Kamenwa R.W., Limbe M.S., Okumu M.O., Macharia W.M. (2021). Prevalence of vitamin D deficiency in exclusively breastfed infants at a tertiary healthcare facility in Nairobi, Kenya. Arch. Endocrinol. Metab..

[53] Ncayiyana J.R., Martinez L., Goddard E., Myer L., Zar H.J. (2021). Prevalence and Correlates of Vitamin D Deficiency among Young South African Infants: A Birth Cohort Study. Nutrients.

[54] Amukele T.K., Soko D., Katundu P., Kamanga M., Sun J., Kumwenda N.I., Taha T.E. (2013). Vitamin D levels in Malawian infants from birth to 24 months. Arch. Dis. Child..

[55] Okonofua F., Houlder S., Bell J., Dandona P. (1986). Vitamin D nutrition in pregnant Nigerian women at term and their newborn infants. J. Clin. Pathol..

[56] Pfitzner M.A., Thacher T.D., Pettifor J.M., Zoakah A.I., Lawson J.O., Isichei C.O., Fischer P.R. (1998). Absence of vitamin D deficiency in young Nigerian children. J. Pediatr..

[57] Donkor W.E.S., Mbai J., Sesay F., Ali S.I., Woodruff B.A., Hussein S.M., Mohamed Mohamud K., Muse A., Said Mohamed W., Muse Mohamoud A. (2022). Risk factors of stunting and wasting in Somali pre-school age children: Results from the 2019 Somalia micronutrient survey. BMC Public Health.

[58] Neary J., Langat A., Singa B., Kinuthia J., Itindi J., Nyaboe E., Ng’anga L.W., Katana A., John-Stewart G.C., McGrath C.J. (2022). Higher prevalence of stunting and poor growth outcomes in HIV-exposed uninfected than HIV-unexposed infants in Kenya. AIDS.

[59] Phiri M., Mulemena D., Kalinda C., Odhiambo J.N. (2022). Contextual factors and spatial trends of childhood malnutrition in Zambia. PLoS ONE.

[60] Bitew F.H., Sparks C.S., Nyarko S.H., Apgar L. (2023). Spatiotemporal Variations and Determinants of Under-Five Stunting in Ethiopia. Food Nutr. Bull..

[61] Ahmed K.Y., Ross A.G., Hussien S.M., Agho K.E., Olusanya B.O., Ogbo F.A. (2023). Mapping Local Variations and the Determinants of Childhood Stunting in Nigeria. Int. J. Environ. Res. Public Health.

[62] Luzingu J.K., Stroupe N., Alaofe H., Jacobs E., Ernst K. (2022). Risk factors associated with under-five stunting, wasting, and underweight in four provinces of the Democratic Republic of Congo: Analysis of the ASSP project baseline data. BMC Public Health.

[63] Katoch O.R. (2022). Determinants of malnutrition among children: A systematic review. Nutrition.

[64] Baroncelli S., Galluzzo C.M., Liotta G., Andreotti M., Orlando S., Ciccacci F., Mphwere R., Luhanga R., Sagno J.B., Amici R. (2021). HIV-exposed infants with EBV infection have a reduced persistence of the immune response to the HBV vaccine. AIDS Res. Ther..

[65] Baroncelli S., Galluzzo C.M., Liotta G., Andreotti M., Jere H., Luhanga R., Sagno J.B., Ciccacci F., Orlando S., Amici R. (2021). Dried blood spots for the quantitative evaluation of IgG isotypes and correlation with serum samples in HIV-exposed uninfected (HEU) infants. J. Immunol. Methods.

[66] Baroncelli S., Galluzzo C.M., Orlando S., Mphwere R., Kavalo T., Luhanga R., Amici R., Floridia M., Andreotti M., Scarcella P. (2022). Dynamics of SARS-CoV-2 exposure in Malawian infants between February 2020 and May 2021. J. Clin. Virol. Plus..

[67] Baroncelli S., Galluzzo C.M., Orlando S., Mphwere R., Kavalo T., Luhanga R., Amici R., Floridia M., Andreotti M., Ciccacci F. (2022). Immunoglobulin G passive transfer from mothers to infants: Total IgG, IgG subclasses and specific antipneumococcal IgG in 6-week Malawian infants exposed or unexposed to HIV. BMC Infect. Dis..

